# Surgical treatment of spinal tenosynovial giant cell tumor: Experience from a single center and literature review

**DOI:** 10.3389/fonc.2022.1063109

**Published:** 2023-01-17

**Authors:** Shiliang Cao, Liang Jiang, Shaomin Yang, Zhongjun Liu, Feng Wei, Xiaoguang Liu

**Affiliations:** ^1^ Department of Orthopaedics, Peking University Third Hospital, Beijing, China; ^2^ Engineering Research Center of Bone and Joint Precision Medicine, Peking University, Bejing, China; ^3^ Beijing Key Laboratory of Spinal Disease Research, Peking University, Beijing, China; ^4^ Department of Interventional Medicine, China Japan Friendship Hospital, Beijing, China; ^5^ Pathology Department, Peking University Third Hospital, Beijing, China

**Keywords:** tenosynovial giant cell tumor (TGCT), spine, resection, treatment, review

## Abstract

**Introduction:**

Spinal tenosynovial giant cell tumor (TGCT) is a rare benign primary spinal tumor with aggressive behavior. The treatment strategy and prognosis of spinal TGCT remain unclear. This retrospective study aimed to evaluate the effectiveness of surgical treatment of spinal TGCT.

**Methods:**

We enrolled 18 patients with spinal TGCT who underwent surgical treatment in our hospital between January 2002 and January 2021. Additionally, we reviewed 72 cases of spinal TGCT with surgical treatment reported in the previous literature. Therefore, a total of 90 cases of spinal TGCT were evaluated for their clinical characteristics, surgical details, radiotherapy, and prognosis.

**Results:**

In terms of the extent of resection, 73 cases (81.1%) underwent gross total resection (GTR), and 17 cases (18.9%) underwent subtotal resection (STR). Regarding the technique of GTR, 12 cases (16.7%) underwent en bloc resection, while 60 cases (83.3%) underwent piecemeal resection. During a median follow-up duration of 36 months (range: 3–528 months), 17.8% (16/90) cases experienced local recurrence/progression. The local recurrence/progression rate in cases that underwent GTR was 8.2% (6/73), which was significantly lower than that in cases with STR (58.8%, 10/17) (*p*<0.001). The local recurrence/progression rate of en bloc resection was 8.3% (1/12), and that of piecemeal resection was 8.3% (5/60). Twelve cases underwent perioperative adjuvant radiotherapy, and one (8.3%, 1/12) of them showed disease progression during follow-up. Six recurrent/progressive lesions were given radiotherapy and all of them remained stable in the subsequent follow-up. Eight recurrent/progressive lesions were only treated with re-operation without radiotherapy, and half of them (50.0%, 4/8) demonstrated repeated recurrence/progression in the subsequent follow-up.

**Conclusion:**

Surgical treatment could be effective for spinal TGCT cases, and GTR is the preferred surgical strategy. Piecemeal resection may be appropriate for spinal TGCT cases with an acceptable local recurrence/progression rate. Perioperative adjuvant radiotherapy may reduce the risk of postoperative local recurrence/progression, and radiotherapy plays an important role in the treatment of recurrent/unresectable spinal TGCT lesions.

## 1 Introduction

Tenosynovial giant cell tumor (TGCT), also called pigmented villonodular synovitis (PVST), is a rare soft tissue tumor derived either from the peri-articular, intra-articular synovial membrane, tendon sheath or bursae. The vast majority of TGCT is a benign tumor that may show aggressive behavior, and malignant TGCT is extremely rare (1). TGCT usually affects the extremities, such as the knee joint, hip joint, and fingers. TGCT involving the spine is extremely rare but has occasionally been reported in single or multiple case series in the previous literature. To date, the largest case series of spinal TGCT have been reported by Furlong et al. in 2003 (2) and Motamedi et al. in 2005 (3), with 15 spinal TGCT cases reported by each.

For TGCT located in the extremities, surgical resection is the preferred curative treatment (4). However, TGCT has shown a propensity for local recurrence after surgical resection, especially diffuse TGCT, with a local recurrence rate of up to 10–48% in previous studies (4–8). Therefore, gross total resection (GTR) with postoperative radiotherapy has been recommended for TGCT to reduce local recurrence (9). Due to the special anatomy and functions of the spine, the clinical characteristics, treatment method, and prognosis of spinal TGCT might be different from those of TGCT of the extremities. However, there is limited experience regarding the surgical treatment and prognosis of spinal TGCT due to its rarity.

To summarize the experience of surgical treatment and prognosis of spinal TGCT, we retrospectively reviewed 18 cases of spinal TGCT that were diagnosed and surgically treated in our hospital over the past 20 years. We also reviewed and compared the 72 spinal TGCT cases reported in previous literature. Finally, we present the clinical information, surgical regimens, radiotherapy, and follow-up outcomes of these combined 90 cases.

## 2 Methods

### 2.1 General information of the hospital patients

This study was approved by ethics committee of our hospital. The requirement for informed consent was waived due to the retrospective nature of this study. After a review of our spinal tumor database, we identified 18 spinal TGCT patients who had undergone surgical treatment at our department between January 2002 and December 2021. Their TGCT diagnosis was confirmed by histopathological examination.

We retrospectively collected and evaluated the hospital charts, surgery information, pathology reports, and imaging information. The following data were collected: age, sex, symptoms, neurologic function, tumor location, radiological features, pathology, surgical procedure, perioperative radiotherapy, outcome, and complications of treatment.

### 2.2 Radiological evaluation and biopsy

Posteroanterior and lateral X-rays of the spine, computed tomography (CT), and magnetic resonance imaging (MRI) were routinely performed preoperatively in all patients to evaluate the lesion. The Enneking Staging system was used to evaluate the lesion based on the CT findings.

All the spinal TGCT patients in our department received percutaneous CT-guided trocar biopsy before surgery, except for one who underwent an unsuccessful surgery at another hospital and was subsequently referred to our department.

### 2.3 Surgical technique

In the previous literature, according to the extent of surgical resection in spinal TGCT cases, the surgical strategy was divided into gross total resection (GTR) and subtotal resection (STR) (2, 10–15). GTR means complete resection without macroscopical residual tumor, includeing en bloc resection and piecemeal resection (intralesional resection). En bloc resection is removing the tumor in one piece without violating the capsule of the tumor based on the WBB (Weinstein-Boriani-Biagnini) classification, or removing the tumor in two pieces with violating the capsule of the tumor by transpedicular osteotomy (16). Piecemeal resection (intralesional resection) begins with careful exposure of the lesion through the normal tissue, and the tumor could be removed as two or more pieces and the capsule could be violated to protect the paraspinal vital structure. STR was defined as incomplete resection with any macroscopical residual tumor, which means curettage or debulking surgery, the capsule of the tumor could be violated with suspicious tumor residual. Internal fixation and fusion could be used to reconstruct the stability of the spine if it is necessary. Perioperative radiotherapy was recommended to spinal TGCT patients according to the experience of the surgeon and oncologist.

### 2.4 Follow-up

Typically, patients underwent X-rays, CT, and MRI scans every 3 months in the first 2 postoperative years, every 6 months in the next 3 years, and annually thereafter. During the follow-up, if the patients had any symptoms indicative of local recurrence, immediate CT and MRI were prescribed. Single photon emission computed tomography (SPECT)and positron emission tomography-computed tomography (PET-CT) were used if the suspected local recurrence could not be confirmed or denied by routine examination.

### 2.5 Literature search

The search terms were taken from MeSH (Medical Subject Headings). MeSH terms and entry terms of “tenosynovial giant cell tumor” or “synovitis, pigmented villonodular” were combined with the MeSH terms and entry terms of “spine” or “cervical spine” or “thoracic spine” or “lumbar spine”. Electrical databases, including PubMed, Embase, Ovid, and Web of Science were searched. The search field was “all field” and the publication time was between January 1980 to January 2022. The study regarding the spinal TGCT cases was first published in 1980 by George M. Kleinman (17).

### 2.6 Study selection and data extraction

The inclusion criteria were as follows: pathological diagnosis of spinal TGCT, surgical treatment, case report, case series report, and retrospective, prospective, and observation research. The exclusion criteria were as follows: unclear treatment or follow-up information in the literature, less than 12 months follow-up period [if the local recurrences were documented in less than 12 months postoperatively, these cases were still included in the study (18, 19)], full-text could not be obtained, conference abstract, review, meta-analysis, book, comment, and letter. After removing duplicate studies, screening the titles and abstracts, and reading full texts, a total of 76 studies were included.

**Figure 1:**
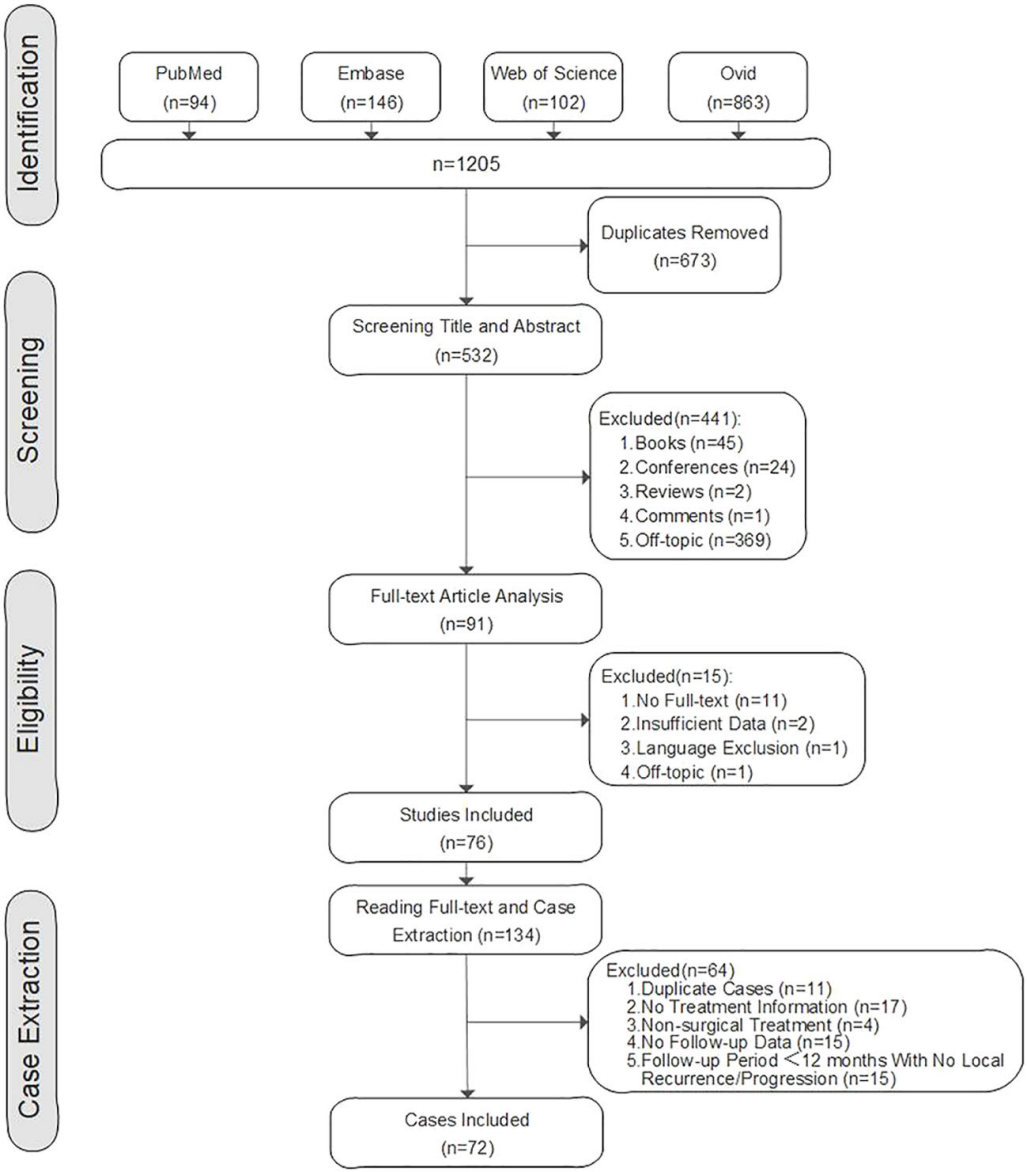
Flow Diagram of Literature Group.

Among 134 spinal TGCT reported in these studies, a total of 62 cases were excluded, including 17 cases with no treatment information, 11 duplicated cases, four cases with non-surgical treatment, 15 cases with no follow-up data, and 15 cases with a follow-up period of less than 12 months without documentation of local recurrence or progression. Finally, 72 spinal TGCT cases with surgical treatment from previous studies were included in the literature group (**Figure 1**).

The demographic information, clinical characteristics, tumor location, surgical information, adjuvant therapy, outcomes, complications, pathology reports, follow-up data, and imaging information of spinal TGCT cases in the previous literature were extracted and summarized.

### 2.7 Statistical analysis

Continuous variables were presented as means and standard deviation (mean ± SD), while discrete variables were presented as frequency counts and percentages. Based on the sample size and expected value, the chi-square test or Fisher test was used to analyze the categorical variables. Statistical significance was set at *p*<0.05. The statistical analysis was performed by SPSS (version 20.0; SPSS, Inc., Chicago, IL).

## 3 Results

### 3.1 Demographic and clinical information

In our spinal TGCT group, there were 18 cases who underwent surgical treatment. All cases were followed-up with a median follow-up period of 67 (range: 12–228) months (**Table 1**).

**Table 1:** Treatment and Outcomes of Spinal tenosynovial giant cell tumor in Our Group.

No.	Gender	Age	Symptom	Frankel Grade	Location	WBB	Surgery	Radiotherapy	Follow-up Months	Last Status
1	F	22	Mass	E	C1-2	A-D/7-12	GTR/PR	Pre-op	228	NED
2	M	30	P	E	C4-5	A-D/9-12	GTR/PR	No	180	NED
3	F	37	P+ND	D	C5-7	A-D/1-5,12	GTR/PR	No	118	NED
4	F	44	P+ND	D	C1-3	A-D/1-6	STR	after recur	102	SWD
5	M	22	P+ND	D	T11	A-D/4-12	GTR/En bloc	No	60	NED
6	M	46	P	E	T9	A-D/2-8	GTR/En bloc	after recur	141	SWD
7	M	28	P+ND	D	C4-6	A-D/1-2,5-12	STR	after recur	27	SWD
8	F	32	P	E	C1-2	A-D/2-8	STR	after recur	72	SWD
9	M	29	P	E	C1-2	A-D/1,6-12	GTR/PR	Post-op	84	NED
10	M	25	P+ND	D	T7-8	A-D/1-2,9-12	GTR/PR	Post-op	120	NED
11	F	45	P+ND	D	C1-2	A-D/1-10	GTR/PR	No	180	NED
12	F	47	P+ND	E	C4-6	A-D/4-11	GTR/PR	Post-op	62	NED
13	F	26	P+ND	D	T2-3	A-D/1,9-12	GTR/PR	No	39	NED
14	F	38	No	E	C1	A-C/1-2,11-12	GTR/PR	Post-op	36	NED
15	M	49	ND	D	C1-2	A-D/1-5	GTR/PR	Post-op	48	NED
16	F	36	P+ND	E	C3-5	A-D/8-12	GTR/PR	No	28	NED
17	M	32	P	E	T1,2	A-D/1-6,12	GTR/PR	No	12	NED
18	F	17	P+ND	D	C6-7	A-D/10-11	GTR/PR	Post-op	12	NED

F, female; M, male; P, local pain; ND, neurological deficit; S3, stage 3; GTR, gross total resection; STR, subtotal resection; PR, piecemeal resection; NED, no evidence of disease; SWD, survival with disease.

In the literature group, there were 72 spinal TGCT cases were enrolled. Their median follow-up period was 28.5 (range: 3–528) months.

Information on sex, age, symptoms, Frankel grade and location, pathological type, and Enneking staging of lesions is summarized in **Table 2**. There was a significant difference in the location of the lesions between our group and the literature group (*p*=0.033).

**Table 2:** Demographic and Clinical Characteristics.

	Our group	Literature group	Total	*p-*value
Case Number	18	72	90	/
Gender (Male)	8 (44.4)	33 (46.5)	41 (46.1)	0.877
Mean age at operation(years)	33.6 ± 9.7	39.2 ± 16.5	38.1 ± 15.5	0.175
Location of Lesion				
Upper Cervical(C1-2)	6 (33.3)	9 (12.5)	15 (16.7)	0.033^a^
Subaxial Cervical(C3-7)	7 (38.9)	25 (34.7)	32 (35.6)	
Thoracic	5 (27.8)	17 (23.6)	22 (24.4)	
Lumbar	0 (0)	19 (26.4)	19 (21.1)	
Sacrum	0 (0)	2 (2.8)	2 (2.2)	
Symptoms				
Local Pain	15 (83.3)	45 (77.6)	60 (78.9)	0.723^a^
Neurological Impairment	11 (61.1)	38 (65.5)	49 (64.5)	
Local mass	1 (5.6)	1 (1.7)	2 (2.6)	
Asymptomatic	1 (5.6)	6 (10.3)	7 (9.2)	
Frankel Grade				
B	0 (0)	1 (5.9)	1 (3.8)	0.178^a^
C	0 (0)	5 (29.4)	5 (19.2)	
D	9 (100)	11 (64.7)	20 (76.9)	
Pathological Types				
Benign	18 (100)	70 (97.2)	88 (97.8)	1.000^a^
Malignant	0 (0)	2 (2.8)	2 (2.2)	
Enneking Staging of Benign TGCT				
S1	0 (0)	3 (10.3)	3 (6.4)	0.092^a^
S2	0 (0)	5 (17.2)	5 (10.6)	
S3	18 (100)	21 (72.4)	39 (83.0)	
Follow-up months	67 [12-228]	28.5 [3-528]^b^	36 [3-528]	/

Data are presented as number (percentage).

^a^Fisher exact test.

^b^There was one case had local recurrence in 3 months after the index surgery without any further information of later follow-up (Hsieh, 2012), another case had distant metastasis and died 6 months later after surgery (Li, 2020).

### 3.2 Surgical technique

In our spinal TGCT group of 18 cases, 15 (83.3%) cases underwent successful GTR, while three (16.7%) cases underwent STR because the lesions were adherent to vital structures (such as the dural sac, and vertebral artery) and difficult to detach. In the 15 GTR cases, 13 (86.7%) cases underwent piecemeal resection, while two (13.3%) underwent en bloc resection (total en bloc spondylectomy).

In the literature group of 72 cases, 58 (80.6%) cases underwent GTR, and 14 (19.4%) underwent STR. Among the GTR cases, 47 (82.5%) cases underwent piecemeal resection, while 10 (17.5%) underwent en bloc resection, and one resection technique was not available due to limited information. There was no significant difference in the surgical technique between the two groups (**Table 3**).

**Table 3:** Extent of Resection and surgical technique.

	Our group	Literature group	Total	*p-*value
Extent of Resection				
GTR	15	58	73	1.000^a^
STR	3	14	17	
Surgical technique				
En bloc	2	10	12	1.000^a^
Piecemeal Resection	13	47	60	

In previous literature, one case without sufficient surgical information to judge whether en bloc resection or piecemeal resection.

^a^Continuity Correction Chi-Square Test.

Among all the 90 cases of spinal TGCT, 73 (81.1%) underwent GTR, and 17 (18.9%) underwent STR. Of all GTR cases, 12 (16.7%) cases underwent en bloc resection and 60 (83.3%) underwent piecemeal resection.

### 3.3 Local recurrence or disease progression

**Figure 2:**
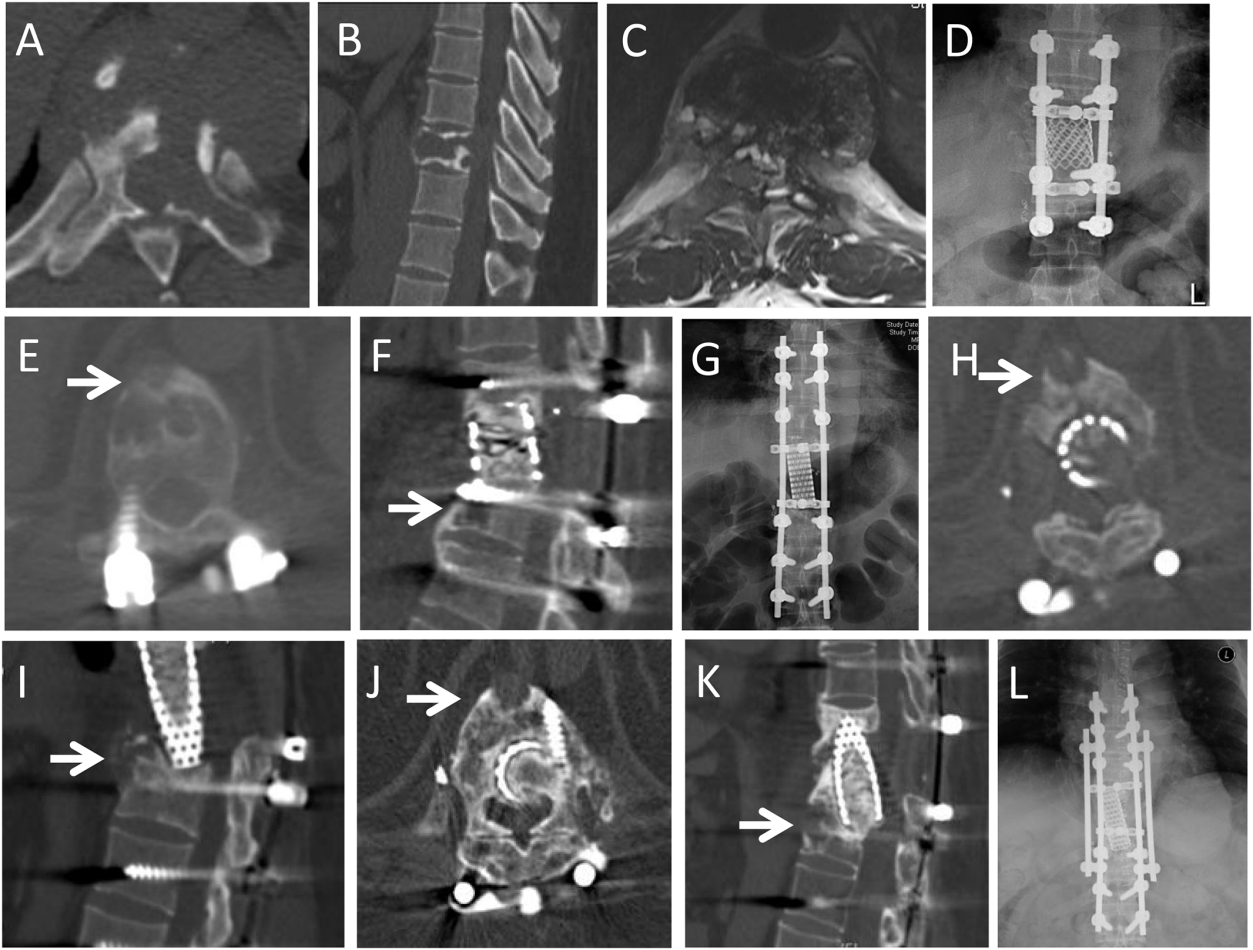
A 46-year-old male patient presented with back pain for one month. CT and MRI showed an osteolytic lesion in the T9 vertebra. He underwent GTR (en bloc vertebrectomy of T9). At 37-month follow-up, he complained of back pain for 3 months, and CT and MRI demonstrated local recurrence at T10 vertebral body (arrows in E,F). He underwent GTR (en bloc vertebrectomy of T10). At 19 months after second operation, he had a sudden back pain and X-ray showed that rod fracture. He underwent the third operation to replace the broken titanium rod with cobalt-chromium-molybdenum rod. At 8 months after third operation, the CT and MRI revealed the local recurrence at anterior part of T11 vertebral body (arrows in H,I). He underwent radiotherapy but refused further surgery. The recurrent lesion was stable in subsequent 52 months follow-up (arrows in J,K), but the rob fractured again. He had to underwent forth operation to replace the rod. **(A,B)**: Preoperative axial and sagittal CT scans revealed the osteolytic lesion extended from the T9 vertebral body to the left pedicle and transverse process; **(C)**: T2-weighted axial MRI scans; **(D)**: Posteroanterior X-ray after first operation; **(E,F)**: Axial and sagittal CT scans at 37-month follow-up after the first operation; **(G)**: Posteroanterior X-ray after second operation; **(H,I)**: Axial and sagittal CT scans 8 month later after third operation; **(J,K)**: Axial and sagittal CT scans in 44th month after radiotherapy; **(L)**: Posteroanterior X-ray after fourth operation.

**Figure 3:**
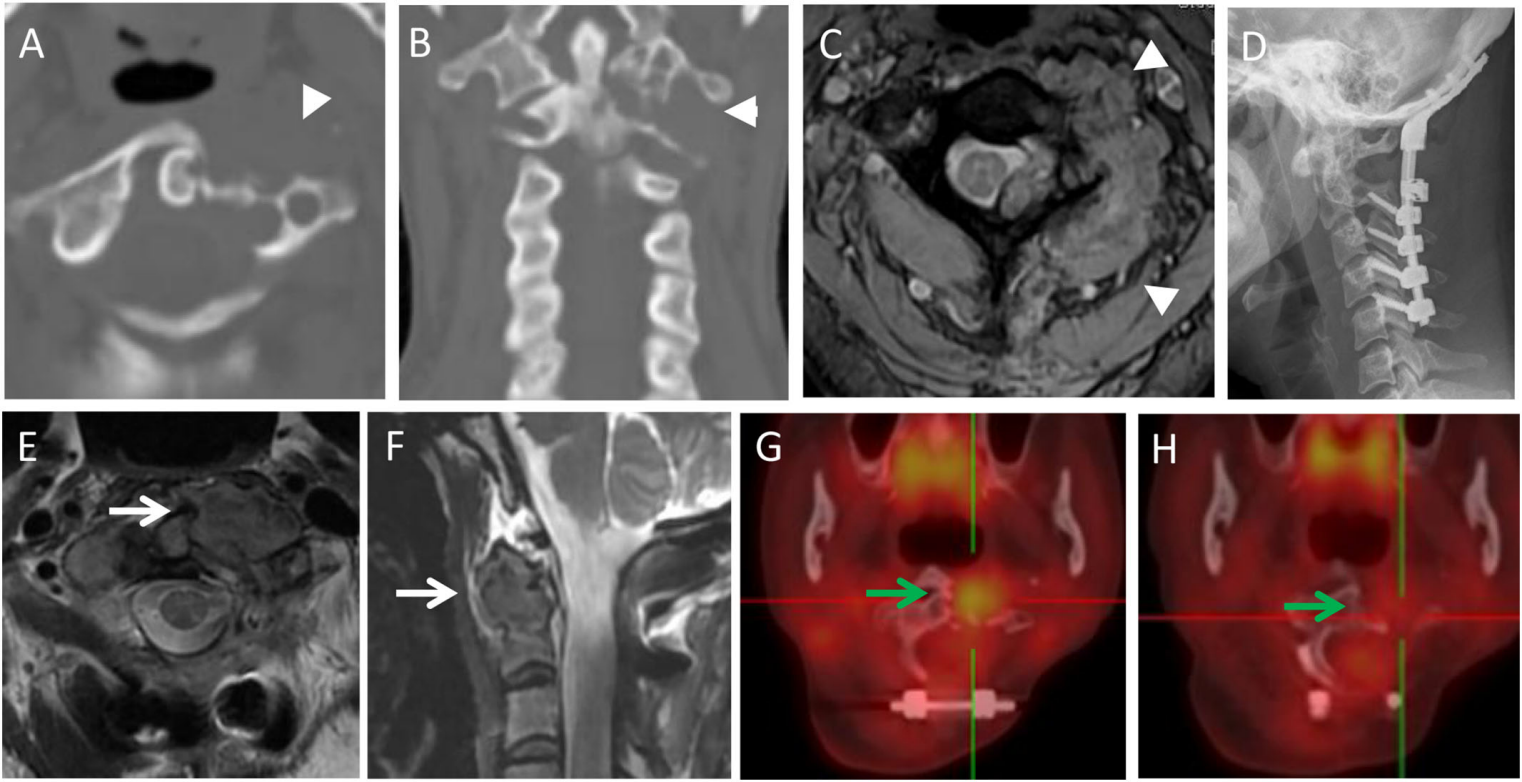
A 44-year-old female with neck pain for five months, an osteolytic lesion was located in the C1-2 (arrowheads). She underwent STR because of vertebral artery invasion. In 6th month follow-up, the MRI scans revealed the local recurrence with a soft tissue mass (white arrows). She underwent radiotherapy but refused further operation. In the subsequent 96 months follow-up, the lesion was stable and repeated PET-CT revealed the reduced uptake of the lesion (green arrows). **(A,B)**: Preoperative axial and coronal CT scans; **(C):** Multiecho gradient-echo sequence axial MRI scan; **(D)**: Postoperative X-ray; **(E,F)**: T2-weighted axial and sagittal MRI scans in 6th month follow-up; **(G)**: PET-CT in 18-month follow-up after radiotherapy (SUV max 6.0); **(H)**: PET-CT in the 90-month follow-up after radiotherapy (SUV max 2.1).

**Figure 4:**
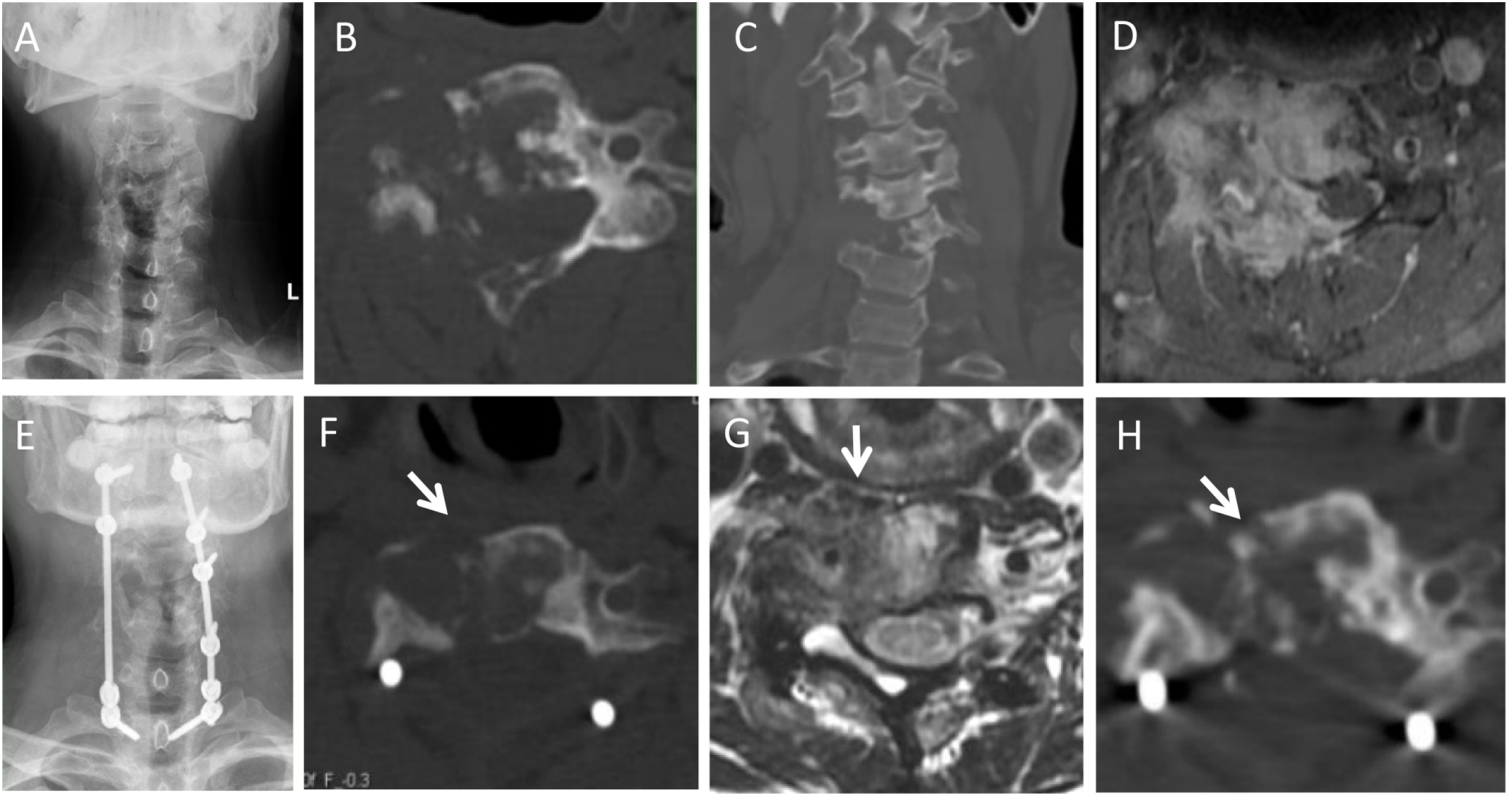
A 28-year-old male with neck pain for 12 months, worsened and accompanied with radiating pain in right upper extremity, shoulder and back, numbness in right hand for six months. Preoperative imaging examination showed a irregular and osteolytic lesion located in C4-6 with scoliosis. GTR was panned, but he underwent STR for strong adhesion between tumor and dural sac, which was revealed during the operation. CT and MRI scans revealed soft tissue mass and osteolytic lesion of local progression at 3-month follow-up after surgery (arrows in **F,G**). He underwent radiotherapy. The lesion was stable with partial osteogenesis in 24-month follow-up after radiotherapy with new bone (arrow in **H**). **(A)**: Preoperative X-ray; **(B,C)**: Preoperative axial and coronal CT scans; **(D)**: T1-weighted axial contrast enhanced MRI scan; **(E)**: Postoperative X-ray; **(F)**: Axial CT scan at 3-month follow-up after surgery; **(G)**: T2-weighted axial MRI scan in 3rd month after surgery; **(H)**: Axial CT scan at 24-month follow-up after radiotherapy.

**Figure 5:**
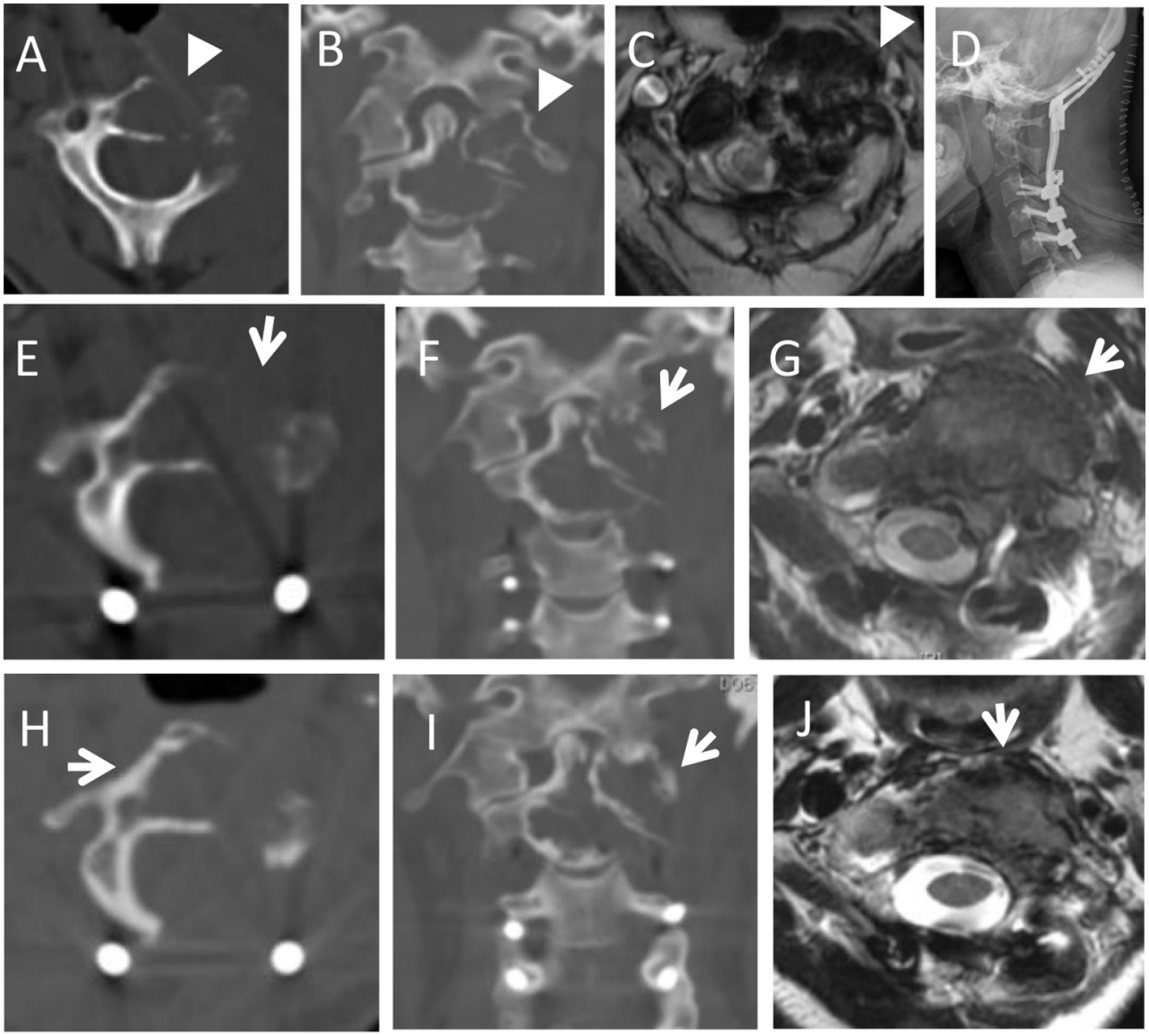
A 32-year-old female with neck pain for four months, and worsened in the following 2 months. Preoperative imaging examination showed an osteolytic lesion was located in the C1-2 (arrowheads in **A, B**). She underwent STR because of lesion adhesion to the vertebral artery. In 3-month follow-up, CT and MRI scans revealed local progression with soft tissue mass (arrows in **E,F,G**). She underwent radiotherapy. And at the 69-month follow-up, the lesion was stable and the volume of soft tissue mass was reduced with slight new bone around the osteolytic lesion (arrows in **H,I,J**). **(A,B)**: Preoperative axial and coronal CT scans; **(C)**: T2-weighted axial MRI scans; **(D)**: Postoperative X-ray; **(E,F)**: Axial and coronary CT scans in 3rd month after surgery; **(G)**: T2-weighted axial MRI scan at the 3-month follow-up after surgery; **(H–J)**: Axial and coronary CT scans, and T2-weighted axial MRI scan at the 69-month follow-up after radiotherapy.

In our spinal TGCT group, 22.2% (4/18) cases demonstrated local recurrence/progression (**Figures 2**–**5**), while 16.7% (12/72) cases had local recurrence/progression in the literature group (*p*=0.836). On combining the data from the two groups, the local recurrence/progression rate among the spinal TGCT cases was 17.8% (16/90).

In all 90 cases, 8.2% of GTR cases (6/73) and 58.8% of STR cases (10/17) experienced local recurrence/progression; however, there was a significant difference in the local recurrence/progression rate (*p*<0.001).

One case (8.3%, 1/12) with en bloc resection and five cases (8.3%, 5/60) with piecemeal resection showed local recurrence/progression (*p*=1.000).

### 3.4 Complications

In our spinal TGCT group, a total of 10 (10/18, 55.6%) cases experienced surgical complications.

Among the GTR cases, surgical complications were observed in 60% (9/15) of the cases, including two cases with prolonged postoperative endotracheal intubation, two with a transient neurological deficit (one after en bloc resection), one with submandibular gland injury, one with an intraoperative dural tear, one with pleural rupture and effusion, and one with repeated internal fixation failure (en bloc resection, Case 6, **Figure 2**).

There was only one (1/3, 33.3%) case with a surgical complication (transient neurological deficit) among the STR cases.

### 3.5 Radiotherapy

In our spinal TGCT group, 38.9% (7/18) cases had perioperative adjuvant radiotherapy (one preoperative and six postoperative), and none of them (0%) developed local recurrence/progression during the follow-up. In the cases without perioperative adjuvant radiotherapy, four of eleven (36.4%) cases had local recurrence/progression (*p*=0.220).

In the literature group, five cases underwent perioperative adjuvant radiotherapy (one preoperative and four postoperative). Four cases demonstrated no local recurrence or progression during the follow-up. One case with postoperative adjuvant radiotherapy had local recurrence and suspicious malignant transformation of the tumor 15 months after STR.

### 3.6 Treatment of lesions with recurrence/progression

There were 14 local recurrent/progressive lesions requiring further treatment (including two repeated recurrent lesions). Among them, six lesions were treated with radiotherapy or re-operation combined with radiotherapy, and none of them showed repeated local recurrence/progression during the subsequent follow-up. The other eight lesions were treated with only re-operation, and four of them (50.0%) had repeated local recurrence or disease progression.

## 4 Discussion

TGCT is a soft tissue tumor, which rarely occurs in the spine, and the clinical characteristics and treatment of spinal TGCT are still controversial. This study retrospectively analyzed the clinical characteristics, surgical outcomes, radiotherapy, and prognosis of 18 spinal TGCT cases who underwent surgical treatment in our hospital as well as 72 spinal TGCT cases reported in the previous literature. Among the total 90 cases, the overall postoperative local recurrence/progression rate was 17.8%, while the local recurrence/progression rate of patients who underwent GTR (8.2%) was significantly lower than that of those who underwent STR (58.8%). Furthermore, radiotherapy may reduce the risk of postoperative local recurrence/progression of spinal TGCT, and contribute to the control of recurrent/progressive lesions.

TGCT presents a wide range of histopathological manifestations, which is composed of varying proportions of histiocytic cells, multinucleated giant cells, lymphocytes, foam cells, and haemosiderin. The stroma of TGCT shows varying degrees of collagenation. Sometimes it is difficult to distinguish TGCT from giant cell tumor (GCT) of bone. If no H3F3 gene mutation is detected, it may help to exclude the diagnosis of GCT (20). The vast majority of TGCT is a benign tumor and malignant TGCT is rare and has some specific histological appearance: diffuse nuclear pleomorphism, prominent nucleoli, high nucleus-to-cytoplasmic ratio, increased mitotic, focal necrosis, decreased intercellular adhesion, lack of multinucleated giant cells and invasive growth pattern (21). Malignant TGCT is very rare in spinal TGCT, and only 2 cases of malignant spinal TGCT have been reported in the previous literature (22, 23), both have had local recurrence after surgery.

Surgical treatment is the preferred treatment for TGCT. However, the postoperative local recurrence rate of TGCT is high due to its aggressive nature and is reportedly 8.5–48.0% for TGCT in the extremities (4, 6, 7, 24, 25). Furlong et al. reported nine spinal TGCT cases with surgical treatment and follow-up, among which 44.4% (4/9) had a local recurrence during the follow-up period of 4 months to 9 years (2). Giannini et al. evaluated 11 spinal TGCT cases who underwent a surgical treatment and found that three cases (27.3%) had a local recurrence during a follow-up of 6 months to 120 months (median follow-up: 42 months) (10). In the present study, the local recurrence/progression rate was 17.8% (16/90) for all spinal TGCT cases. The local recurrence/progression rates in our group and literature group were 22.2% (4/18) and 16.7% (12/72), respectively.

**Figure 6:**
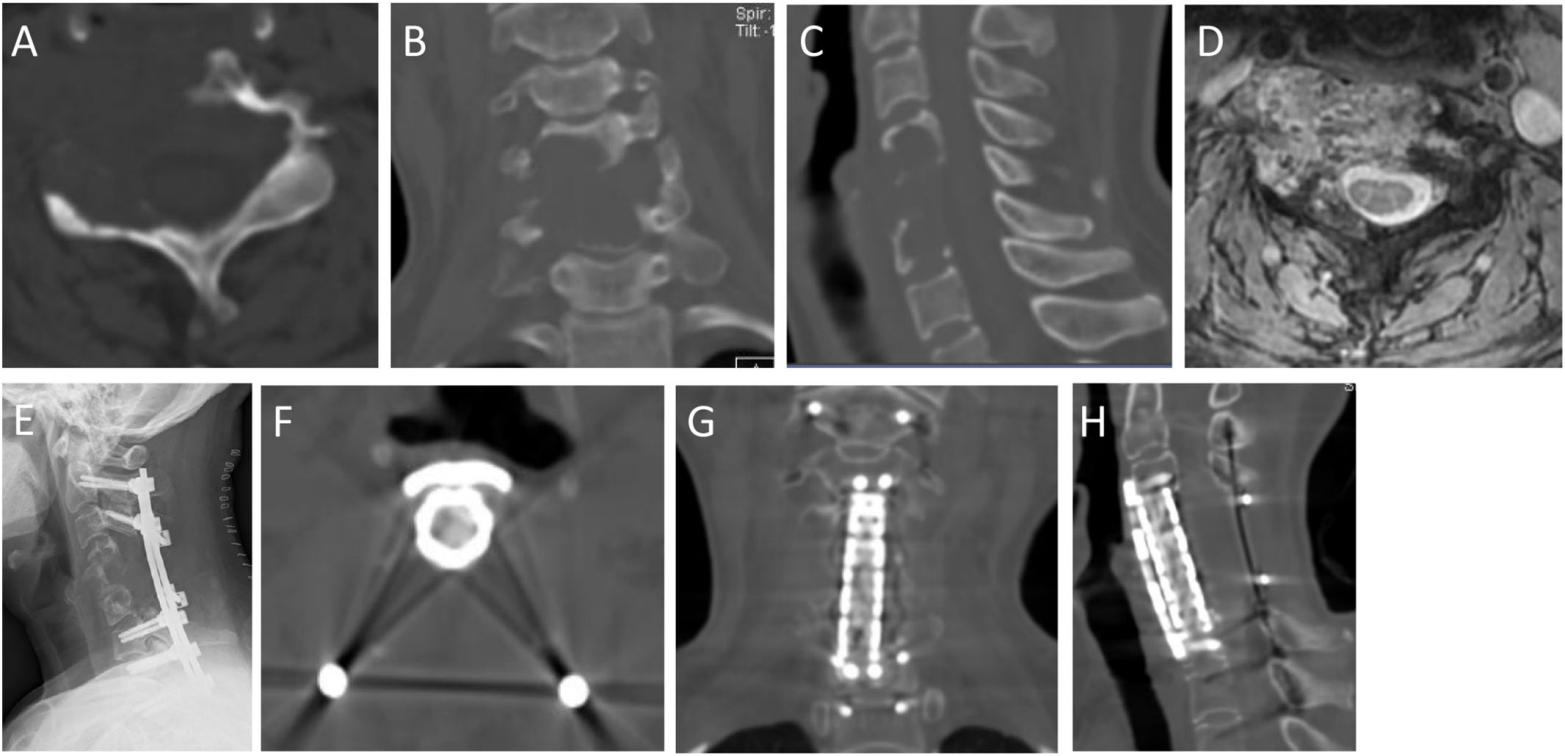
A 47-year-old female with neck pain for 12 months, accompanied with pain and numbness of left upper extremity for the recent 1 month. Preoperative imaging examination showed an osteolytic lesion was in the C4-6. She underwent GTR (piecemeal spondylectomy). At 62-month follow-up, she was symptom free. **(A,B,C)**: Preoperative axial, coronal and sagittal CT scans; **(D)**: T2-weighted axial MRI scans; **(E)**: Postoperative X-ray; **(F–H)**: Axial, coronary and sagittal CT scans in 62th month after surgery.

GTR is usually the appropriate choice for TGCT, which is aggressive and has a propensity for local recurrence (2, 14, 26–28) (**Figure 6**). Macroscopically complete and extensive resection is very important for surgical treatment of TGCT located in the extremities and was found to be associated with better local control in a previous study (29). Furlong et al. reported six spinal TGCT cases who underwent GTR, and none of them demonstrated local recurrence during a median follow-up of 66 months (range: 36–120 months) (2). Giannini et al. investigated 10 spinal TGCT cases who underwent GTR, and two of them (20.0%, 2/10) had a local recurrence during the follow-up of 6 to 118 months (10). Similarly, Li et al. found that out of 11 spinal TGCT cases who underwent GTR, none had recurrence/progression during a median follow-up period of 17 months (range: 4–85) (30). In our study, the local recurrence/progression rate among spinal TGCT cases who underwent GTR was 8.2% (6/73), which was acceptable and significantly lower than that of cases who underwent STR (58.8%, 10/17).

En bloc resection is recommended for spinal tumors to minimize the risk of local recurrence, especially in aggressive lesions (Enneking stage 3). However, it is a demanding technique, especially for lesions involving vital structures (vertebral artery, dural sac, et al). Piecemeal resection was the main surgical technique used for spinal TGCT cases in the present study, with 83.3% (60/72) GTR cases undergoing piecemeal resection, and only 12 of 72 (16.7%) cases undergoing en bloc resection. During follow-up, the local recurrence/progression rate of piecemeal resection was 8.3% (5/60), and there was no significant difference when compared to cases that underwent en bloc resection. The local recurrence/progression rate of piecemeal resection was found to be acceptable (**Figure 6**).

Perioperative adjuvant radiotherapy has been recommended for TGCT in the extremities to reduce the risk of local recurrence in some previous studies (24, 31, 32). In our study, 12 spinal TGCT cases underwent perioperative adjuvant radiotherapy, and none of them had a local recurrence. Besides, six cases with recurrent/progressive lesions underwent radiotherapy, and all of them were stable in the subsequent follow-up. In contrast, eight cases with recurrent/progressive lesions only underwent reoperation without radiotherapy, and half of them (50.0%, 4/8) had repeated recurrence/progression in the subsequent follow-up. Therefore, we recommend perioperative adjuvant radiotherapy for spinal TGCT to reduce the risk of postoperative local recurrence/progression, especially when there was a suspected residual tumor. Radiotherapy could contribute to the local control of recurrent/progressive spinal TGCT lesions. In addition, there was limited experience with preoperative radiotherapy in spinal TGCT patients in our department and related reports were also rare. In this study, only two patients with spinal TGCT underwent preoperative radiotherapy. One was the first spinal TGCT patient treated in our department, and the other was reported by Graham (33), who received preoperative radiotherapy because the patient initially refused surgery and was misdiagnosed. There was no local recurrence in either of them at the last follow-up.

There are some limitations of our study. First, since this was a retrospective single-center study, selection bias was inevitable. Moreover, the sample size was relatively small, and although we collected the information from previous case reports and case series for comparative and combined analyses, the data quality of the literature was difficult to guarantee. However, to our knowledge, this is the largest study on spinal TGCT. In the future, prospective, multicenter studies on spinal TGCT with a large sample size are needed.

In conclusion, our results revealed that surgical treatment for spinal TGCT was effective and GTR was the preferred surgical technique with a lower local recurrence/progression rate compared to STR. Piecemeal resection may be the appropriate surgical technique for spinal TGCT. Perioperative adjuvant radiotherapy may reduce the risk of postoperative local recurrence/progression, and radiotherapy plays an important role in the treatment of recurrent/unresectable spinal TGCT lesions.

## Data availability statement

The raw data supporting the conclusions of this article will be made available by the authors, without undue reservation.

## Ethics statement

The studies involving human participants were reviewed and approved by Peking University Third Hospital Medical Science Research Ethics Committee. Written informed consent from the participants’ legal guardian/next of kin was not required to participate in this study in accordance with the national legislation and the institutional requirements.

## Author contributions

CS contributed to the acquisition and interpretation of the data, wrote the initial draft of the paper, and gave final approval of the manuscript. WF, LX, and LZ contributed to the surgical procedure. YS contributed to the pathological suggestion. JL contributed to the conception and design of the study and the surgical procedure, revised the initial drafts of the paper, and gave approval of the final manuscript. All authors read and approved the final manuscript.
